# Numerical algorithm for environmental/economic load dispatch with emissions constraints

**DOI:** 10.1038/s41598-024-53291-x

**Published:** 2024-02-09

**Authors:** Christos Bakos, Angelos Giakoumis

**Affiliations:** Department of Information and Electronic Engineering, International Hellenic University (IHU), Sindos, Thessaloniki, Greece

**Keywords:** Mathematics and computing, Electrical and electronic engineering

## Abstract

This paper presents a numerical algorithm for environmental/economic load dispatch (EELD) with emissions constraints, which takes into account the emissions trading system’s effect on electricity generation cost and is implemented using a Python computer program. The developed program is applied to a power system of six (6) fossil-fueled electricity generating units with NO_x_, SO_2_ and CO_2_ constraints and proved to be significantly beneficial not only for the environment but also for the power company and the consumers. The proposed algorithm uses multi-objective optimization and incorporates both fuel and emissions allowances costs. The schedule of generating units is calculated and the testing of all possible weighting factor combinations with resolution of 0.01 is carried out showing that the proposed algorithm is fast, cost effective and environmentally friendly.

## Introduction

Strong policy action is necessary to curb greenhouse gas (GHG) emissions. Despite its shortcomings, a feature of the climate regime has been its willingness to experiment with a wide range of tools, including decentralized, market-based institutions. Market-based institutions rely on markets to price externalities and change the behavior of firms and individuals. Among such institutions, European Union's Emissions Trading System (EU ETS) is aiming at reducing greenhouse gases emissions. The scheme has been divided into a number of "trading periods". The first ETS trading period lasted three years, from January 2005 to December 2007. The second trading period ran from January 2008 until December 2012, coinciding with the first commitment period of the Kyoto Protocol. The third trading period lasted from January 2013 to December 2020. The fourth phase started in January 2021 and will go until December 2030. The EU commission proposes in its "Fit for 55" package to increase the EU ETS reduction target for 2030 to − 61% versus 2005^1^. Under this context, a modern solution to the Environmental/Economic Load Dispatch (EELD) problem should not disregard the ETS. An EELD should no longer be limited only to scheduling the committed units’ outputs in order to satisfy demand and inequality constraints, while keeping cost and emissions minimum. It should also take into consideration the equivalent cost of emissions associated with the implementation of the ETS.

Numerical algorithms play a crucial role in various scientific subjects providing computational solutions to problems that may not have analytical solutions or are too complex to solve by hand. Very important research work in the field of optimization and numerical algorithms has been published recently^2–9^.

A number of approaches to the EELD problem have been proposed and a comparative study of them is presented by Talaq et al.^10^ and Sudhansu Kumar Mishra et al.^11^. The EELD is formulated as a multi-objective optimization problem, which means that the goal is to accomplish a number of objectives at once. The basic approach towards multi-objective optimization is the minimization of a function that includes the fuel cost as well as emissions quantity^12^. Improved versions of the multi-objective function, together with evaluation methods for the resulting schedules have been proposed. Wang et al.^13^ created an evolutionary algorithm presenting emissions as constraints while Abido^14,15^ presented a multi-objective evolutionary algorithm using strength Pareto and Niched Pareto Genetic Algorithm. Rughooputh et al.^16^ used the NSGA II algorithm which represents a stochastic approach. Other methods that have been proposed utilize heuristic guided genetic algorithms^17^, fuzzy models^18^ and neural networks^19^.

The numerical algorithm proposed in this paper incorporates the Emissions Trading System to the multi-objective optimization problem. The price of NO_x_, SO_2_ and CO_2_ emissions is taken into consideration in addition to the emissions quantity and fuel cost. Thus, the dispatcher has the ability either to calculate a Pareto optimal solution through the minimization of a multi-objective function or to calculate the schedule that results in the lowest overall cost^20^. A Python program was developed in order to automatically provide the dispatcher with the above results.

## Problem formulation

### Fuel cost

In order to approximate the fuel cost in relation to thermal units power output, a quadratic function is often used, such as:

$$F_{i} (P_{i} ) = a_{F,i} P_{i}^{2} + b_{F,i} P_{i} + c_{F,i} \quad \left( {\$ /{\text{h}}} \right),$$

where $F_{i} (P_{i} )$ represents the fuel cost,$P_{i}$ represents the power output and $a_{F,i} ,b_{F,i} ,c_{F,i}$ are the cost coefficients of the ith unit.

### Emissions

The hourly emissions of NO_x_, SO_2_ and CO_2_ can also be described by sets of quadratic functions of the power output of each thermal unit, such as:

$$E_{{NO_{x} ,i}} (P_{i} ) = a_{{NO_{x} ,i}} P_{i}^{2} + b_{{NO_{x} ,i}} P_{i} + c_{{NO_{x} ,i}} \;\left( {{\text{kg}}/{\text{h}}} \right),$$

$$E_{{SO_{2} ,i}} (P_{i} ) = a_{{SO_{2} ,i}} P_{i}^{2} + b_{{SO_{2} ,i}} P_{i} + c_{{SO_{2} ,i}} \quad \left( {{\text{kg}}/{\text{h}}} \right),$$

$$E_{{CO_{2} ,i}} (P_{i} ) = a_{{CO_{2} ,i}} P_{i}^{2} + b_{{CO_{2} ,i}} P_{i} + c_{{CO_{2} ,i}} \;\left( {{\text{kg}}/{\text{h}}} \right).$$

### Constraints

A power system operation is subject to certain constraints. If losses are overlooked for the simplification of the problem, these constraints are:

$$\sum\limits_{i = 1}^{N} {P_{i} } = P_{load} { ,}$$

$$P_{i,\min } \le P_{i} \le P_{i,\max } .$$

The combined power output of the N generating units must equal the load power demand, P_load_, and each unit’s power output, P_i_, must be between predefined upper and lower limits P_i,min_ and P_i,max_.

## Multi-objective optimization

In a realistic scenario, a dispatcher is obliged to value the relative importance of each of the objectives, because not all objectives can be fully achieved in each schedule. To this end, a multi-objective function can be used which includes all objectives and results in a Pareto optimal solution when minimized. This function is as follows:

$$T_{i} \left( {P_{i} } \right) \, = W_{cost} \left( {\frac{{F_{i} \left( {P_{i} } \right) - \sum\limits_{i = 1}^{N} {F_{i} \left( {P_{i} } \right)_{ideal} } }}{{\sum\limits_{i = 1}^{N} {F_{i} \left( {P_{i} } \right)_{non\_ideal} - \sum\limits_{i = 1}^{N} {F_{i} \left( {P_{i} } \right)_{ideal} } } }}} \right) + W_{{NO_{x} }} \left( {\frac{{E_{{NO_{x,i} }} (P_{i} ) - \sum\limits_{i = 1}^{N} {E_{{NO_{x,i} }} (P_{i} )_{ideal} } }}{{\sum\limits_{i = 1}^{N} {E_{{NO_{x,i} }} (P_{i} )_{non\_ideal} } - \sum\limits_{i = 1}^{N} {E_{{NO_{x,i} }} (P_{i} )_{ideal} } }}} \right) + W_{{SO_{2} }} \left( {\frac{{E_{{SO_{2} ,i}} (P_{i} ) - \sum\limits_{i = 1}^{N} {E_{{SO_{2,i} }} (P_{i} )_{ideal} } }}{{\sum\limits_{i = 1}^{N} {E_{{SO_{2,i} }} (P_{i} )_{non\_ideal} } - \sum\limits_{i = 1}^{N} {E_{{SO_{2,i} }} (P_{i} )_{ideal} } }}} \right) + W_{{CO_{2} }} \left( {\frac{{E_{{CO_{2,i} }} (P_{i} ) - \sum\limits_{i = 1}^{N} {E_{{CO_{2,i} }} (P_{i} )_{ideal} } }}{{\sum\limits_{i = 1}^{N} {E_{{CO_{2,i} }} (P_{i} )_{non\_ideal} } - \sum\limits_{i = 1}^{N} {E_{{CO_{2,i} }} (P_{i} )_{ideal} } }}} \right),$$

where $\sum\nolimits_{i = 1}^{N} {F_{i} (P_{i} )}_{ideal} ,\sum\nolimits_{i = 1}^{N} {E_{{NO_{x} ,i}} (P_{i} )_{ideal} } ,\sum\nolimits_{i = 1}^{N} {E_{{SO_{2} ,i}} (P_{i} )_{ideal} } {\text{ and }}\sum\nolimits_{i = 1}^{N} {E_{{CO_{2} ,i}} (P_{i} )_{ideal} }$ represent the overall fuel cost and NO_2_, SO_2_ and CO_2_ emissions respectively, which correspond to single-objective optimization problem optimal solution.$\sum\nolimits_{i = 1}^{N} {F_{i} (P_{i} )_{non\_ideal} } ,\sum\nolimits_{i = 1}^{N} {E_{{NO_{x} ,i}} (P_{i} )_{non\_ideal} } ,\sum\nolimits_{i = 1}^{N} {E_{{SO_{2} ,i}} (P_{i} )_{non\_ideal} } {\text{ and }}\sum\nolimits_{i = 1}^{N} {E_{{CO_{2} ,i}} (P_{i} )_{non\_ideal} }$ represent the overall fuel cost and NO_2_, SO_2_ and CO_2_ emissions respectively, which correspond to single-objective optimization problem worst case scenario solution.

$W_{\cos t} ,W_{{NO_{x} }} ,W_{{SO_{2} }} ,W_{{CO_{2} }}$ represent the weighting factors, the values of which are at the Dispatcher’s will to choose, according to the priorities of the power system.

The multi-objective function is subject to the following constraints:

$$W_{\cos t} + W_{{NO_{x} }} + W_{{SO_{2} }} + W_{{CO_{2} }} = 100\% ,$$

$$\sum\limits_{i = 1}^{N} {F_{i} (P_{i} )}_{ideal} \le \, \sum\limits_{i = 1}^{N} {F_{i} (P_{i} )} \, \le \, \sum\limits_{i = 1}^{N} {F_{i} (P_{i} )}_{non\_ideal} ,$$

$$\sum\limits_{i = 1}^{N} {E_{{NO_{x} ,i}} (P_{i} )_{ideal} } \, \le \, \sum\limits_{i = 1}^{N} {E_{{NO_{x} ,i}} (P_{i} )} \, \le \, \sum\limits_{i = 1}^{N} {E_{{NO_{x} ,i}} (P_{i} )_{non\_ideal} } ,$$

$$\sum\limits_{i = 1}^{N} {E_{{SO_{2} ,i}} (P_{i} )_{ideal} } \; \, \le \, \sum\limits_{i = 1}^{N} {E_{{SO_{2} ,i}} (P_{i} )} \, \le \, \sum\limits_{i = 1}^{N} {E_{{SO_{2} ,i}} (P_{i} )_{non\_ideal} } ,$$

$$\sum\limits_{i = 1}^{N} {E_{{CO_{2} ,i}} (P_{i} )_{ideal} } \, \le \; \, \sum\limits_{i = 1}^{N} {E_{{CO_{2} ,i}} (P_{i} ) \, } \le \; \, \sum\limits_{i = 1}^{N} {E_{{CO_{2} ,i}} (P_{i} )_{non\_ideal} } ,$$

$$\sum\limits_{i = 1}^{N} {P_{i} } = P_{load} ,$$

$$P_{i,\min } \le P_{i} \le P_{i,\max } .$$

## Emissions trading system implementation

The aforementioned method, while very flexible, lacks one parameter that is of vital importance in order to be considered applicable in the European Union. According to the Emissions Trading System, each industrial unit is assigned a certain amount of pollutant emissions (cap) for a certain period. For every ton exceeding the designated cap, the company is obliged to purchase an allowance from an industrial unit that has achieved lower emissions than the cap. Therefore, fuel cost cannot be the only factor to be considered in the solution of the Environmental/Economic Load Dispatch. The additional factor is the total cost (TC), which includes both fuel and emissions allowances cost and can be calculated through the following equation:

$$TC = \sum\limits_{i = 1}^{N} {F_{i} (P_{i} )} + coprice\left( {10^{ - 3} \times \sum\limits_{i = 1}^{N} {E_{{CO_{2} ,i}} (P_{i} )} - cocon} \right),$$

where *TC* is the total cost, *coprice* is the market price for a CO_2_ allowance, *cocon* is the overall designated cap for CO_2_ emissions, of the power system.

The above formula is a realistic one, because it only takes into consideration CO_2_ emissions which are considered to have the greatest impact to the Greenhouse effect. However the member states of EU are moving towards the incorporation of ΝΟ_x_ and SO_2_ emissions into the ETS. In this case, the total cost should be formulated as follows:

$$\begin{gathered} TC = \sum\limits_{i = 1}^{N} {F_{i} (P_{i} )} + coprice\left( {10^{ - 3} \times \sum\limits_{i = 1}^{N} {E_{{CO_{2} ,i}} (P_{i} )} - cocon} \right) + soprice\left( {10^{ - 3} \times \sum\limits_{i = 1}^{N} {E_{{SO_{2} ,i}} (P_{i} )} - socon} \right) \hfill \\ \, + noprice\left( {10^{ - 3} \times \sum\limits_{i = 1}^{N} {E_{{NO_{x} ,i}} (P_{i} )} - nocon} \right){ , } \hfill \\ \end{gathered}$$

where *TC* is the total cost, *noprice, soprice*, *coprice* is the market price for a NO_x_, SO_2_ and CO_2_ allowance, respectively, *nocon*, *socon*, *cocon* is the overall designated caps for NO_x_, SO_2_ and CO_2_ emissions respectively, of the power system.

**Figure 1:**
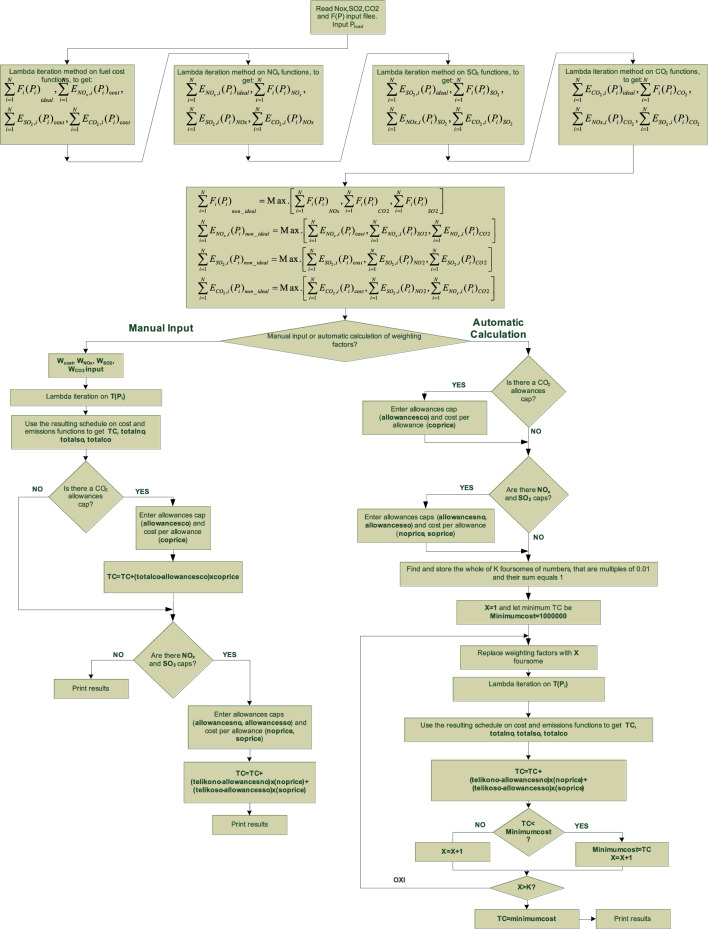
Program flow chart.

In order to assess the effectiveness of the proposed EELD method, a computer program was developed using Python programming language^21^. The flow chart of the developed program is shown in Fig. 1.

Basically, it incorporates two main features:

- Calculation of the environmental/economic load dispatch according to fixed weighting factors chosen by the dispatcher.
- Automatic calculation of the weighting factors resulting in an EELD solution with the lowest possible TC.

## Case study—discussion

In this case study the considered power system consists of six (6) thermal power plants described by the following sets of equations:

Fuel cost ($/h):

$$\begin{gathered} F(P_{1} ) = 0.002035P_{1}^{2} + 8.43205P_{1} + 85.6348{ ,} \hfill \\ F(P_{2} ) = 0.003866P_{2}^{2} + 6.41031P_{2} + 303.7780{ ,} \hfill \\ F(P_{3} ) = 0.002182P_{3}^{2} + 7.42890P_{3} + 847.1484{ ,} \hfill \\ F(P_{4} ) = 0.001345P_{4}^{2} + 8.30154P_{4} + 274.2241{ ,} \hfill \\ F(P_{5} ) = 0.002162P_{5}^{2} + 7.42890P_{5} + 847.1484{ ,} \hfill \\ F(P_{6} ) = 0.005963P_{6}^{2} + 6.91559P_{6} + 202.0258 \, {.} \hfill \\ \hfill \\ \end{gathered}$$

NO_x_ emissions (kg/h):

$$\begin{gathered} F_{21} = 0.006323P_{1}^{2} - 0.38128P_{1} + 80.9019, \hfill \\ F_{22} = 0.006483P_{2}^{2} - 0.79027P_{2} + 28.8249, \hfill \\ F_{23} = 0.003174P_{3}^{2} - 1.36061P_{3} + 324.1775, \hfill \\ F_{24} = 0.006732P_{4}^{2} - 2.39928P_{4} + 610.2535, \hfill \\ F_{25} = 0.003174P_{5}^{2} - 1.36061P_{5} + 324.1775, \hfill \\ F_{26} = 0.006181P_{6}^{2} - 0.39077P_{6} + 50.3808. \hfill \\ \end{gathered}$$

SO_2_ emissions (kg/h):

$$\begin{gathered} F_{31} = 0.001206P_{1}^{2} + 5.05928P_{1} + 51.3778{ ,} \hfill \\ F_{32} = 0.002320P_{2}^{2} + 3.84624P_{2} + 182.2605{ ,} \hfill \\ F_{33} = 0.001284P_{3}^{2} + 4.45647P_{3} + 508.5207{ ,} \hfill \\ F_{34} = 0.110813P_{4}^{2} + 4.97641P_{4} + 165.3433{ ,} \hfill \\ F_{35} = 0.001284P_{5}^{2} + 4.45647P_{5} + 508.5207{ ,} \hfill \\ F_{36} = 0.003578P_{6}^{2} + 4.14938P_{6} + 121.2133 \, {.} \hfill \\ \hfill \\ \end{gathered}$$

CO_2_ emissions (kg/h):

$$\begin{gathered} F_{41} = 0.265110P_{1}^{2} - 61.01945P_{1} + 5080.148{ ,} \hfill \\ F_{42} = 0.140053P_{2}^{2} - 29.95221P_{2} + 3824.770{ ,} \hfill \\ F_{43} = 0.105929P_{3}^{2} - 9.552794P_{3} + 1342.851{ ,} \hfill \\ F_{44} = 0.106409P_{4}^{2} - 12.73642P_{4} + 1819.625{ ,} \hfill \\ F_{45} = 0.105929P_{5}^{2} - 9.552794P_{5} + 1342.851{ ,} \hfill \\ F_{46} = 0.403144P_{6}^{2} - 121.9812P_{6} + 11381.070 \, {.} \hfill \\ \hfill \\ \end{gathered}$$

with the following power outputs constraints:

$$100 \le P_{i} \le 600{\text{ (MW)}}{.}$$

### Total cost (TC) and CO_2_ emissions

**Figure 2:**
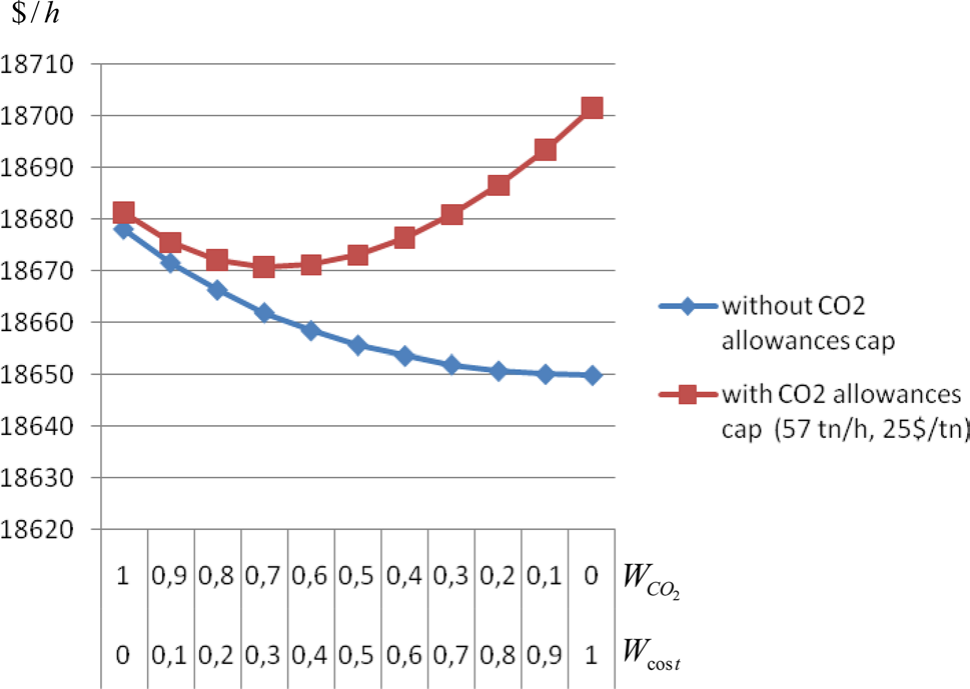
Total power system cost with and without CO_2_ allowances.

The most realistic and up-to-date scenario, is that the only environmental constraint applied to the power system is CO_2_ emissions. The following study focuses on the effect of a certain allowances cap on the total cost using the weighting factors method. The total cost with and without CO_2_ allowances and the total load demand are given in Table 1. Figure 2 shows that when no allowances cap has been set, any increase in W_cost_ will result in a decrease of total cost. This happens because the units that produce cheaper power will be increasingly burdened. However cheaper units are usually emitting larger quantities of pollutants. That means that as W_cost_ increases, CO_2_ emissions increase as well. Thus, when an allowances cap has been set, if too much power is assigned to cheaper and polluting units, the cost for allowances purchases will surpass the gain from smaller fuel cost. In this case, it is found that when W_cost_ = 0.3 and $W_{{CO_{2} }}$ = 0.7 the schedule turns out to be the most economic.

**Table 1:** Total cost with and without CO_2_ allowances and total load demand.

Cost weighting factor (W_cost_)	CO_2_ weighting factor (W_CO2_)	Total cost without CO_2_ allowances ($/h)	Total cost with CO_2_ allowances ($/h)
0	1	18,678.06	18,681.18
0.1	0.9	18,671.56	18,675.35
0.2	0.8	18,666.30	18,672.00
**0.3**	**0.7**	**18,661.90**	**18,670.56**
0.4	0.6	18,658.44	18,670.98
0.5	0.5	18,655.65	18,672.90
0.6	0.4	18,653.58	18,676.30
0.7	0.3	18,651.91	18,680.78
0.8	0.2	18,650.73	18,686.44
0.9	0.1	18,650.11	18,693.37
1	0	18,649.90	18,701.48
Total load demand = 1930 MW			

Significant values are in bold.

Table 2 shows for six (6) different strategies (a) the contribution of each generating unit, (b) the corresponding total cost with and without allowances, (c) the total NO_x_, SO_2_, CO_2_ emissions and (d) the corresponding weighting factors (WFs) values set by the dispatcher. The total load demand for all strategies is kept constant (P_load_ = 1930 MW).

**Table 2:** Generation units contribution, total cost with and without allowances, total NO_x_, SO_2_, CO_2_ emissions and WFs values for different strategies.

Strategies for different WFs combinations (with Wcost decreasing order)	Strategy 1 **W**_**cost**_** = 1** W_NOx_ = 0 W_SO2_ = 0 W_CO2_ = 0	Strategy 2 W_cost_ = 0.7 W_NOx_ = 0.1 W_SO2_ = 0.1 W_CO2_ = 0.1	Strategy 3 W_cost_ = 0.4 W_NOx_ = 0.2 W_SO2_ = 0.2 W_CO2_ = 0.2	Strategy 4 W_cost_ = 0.25 **W**_**NOx**_** = 0.25** W_SO2_ = 0.25 W_CO2_ = 0.25	Strategy 5 W_cost_ = 0.2 W_NOx_ = 0 **W**_**SO2**_** = 0.8** W_CO2_ = 0	Strategy 6 W_cost_ = 0.1 W_NOx_ = 0.1 W_SO2_ = 0.1 **W**_**CO2**_** = 0.7**
Contribution of each generating unit (MW)	P1 = 196.209 P2 = 364.758 P3 = 412.860 P4 = 345.384 P5 = 416.679 P6 = 194.110	P1 = 207.218 P2 = 358.761 P3 = 418.391 P4 = 324.088 P5 = 421.809 P6 = 199.733	P1 = 218.780 P2 = 347.182 P3 = 426.135 P4 = 300.766 P5 = 428.828 P6 = 208.309	P1 = 224.776 P2 = 336.790 P3 = 431.669 P4 = 288.292 P5 = 433.742 P6 = 214.731	P1 = 259.641 P2 = 398.101 P3 = 472.165 P4 = 107.753 P5 = 476.602 P6 = 215.738	P1 = 238.097 P2 = 343.749 P3 = 388.405 P4 = 341.523 P5 = 389.063 P6 = 229.163
Total cost, allowances included (if any) ($/h)	**18,649.9954**	18,699.7360	18,707.7167	18,718.7671	19,032.1111	18,675.8467
Total cost, allowances not included ($/h)	**18,649.9954**	18,651.1727	18,656.7136	18,662.8733	18,756.6654	18,665.6285
Total NO_x_ emissions (kg/h)	2256.4839	2237.2988	2215.9538	**2201.6609**	2619.5510	2283.7562
Total SO_2_ emissions (kg/h)	24,301.0694	22,732.6626	21,133.0659	20,328.2068	**12,517.1471**	24,036.8054
Total CO_2_ emissions (kg/h)	59,063.6938	58,942.5315	59,040.1242	59,235.7524	68,017.8294	**57,408.7304**
Total load demand = 1930 MW						

Significant values are in bold.

### Automatic calculation of weighting factors (WFs)

As it was explained, the dispatcher could meet the total load demand and satisfy the schedule environmental goals by inserting manually the appropriate weighting factors. However, this mode of operation is time consuming and not very efficient. The following study makes use of the automatic feature of the developed software where (a) the values of the weighting factors are changing in a resolution of 0.01 in order to keep the power system running in the most cost-effective way and (b) the dispatcher can rapidly adapt the schedule to the allowances market changes and the total load demand. These advantages of the proposed software are demonstrated below where three (3) different cases were considered.

### Case 1. The impact of variable CO_2_ allowances price on the WFs values

In this case the importance of CO_2_ allowances price variation on the weighting factors values is examined. The CO_2_ allowances prices varied from 0 to 60 $/tn with incremental steps of 2$/tn. As shown in Table 3, the CO_2_ allowances cap was considered constant at 57 tn/h, the total load demand was also constant at 1930 MW and for the remaining pollutants (NO_x_ and SO_2_) no allowances were considered.

**Table 3:** Software settings to check the impact of CO2 allowance price variation on the values of WFs.

P_load_ = 1930 MW	NO_x_	SO_2_	CO_2_
Allowances cap (tn/h)	0	0	57
Allowances price ($/tn)	0	0	0–60

**Figure 3:**
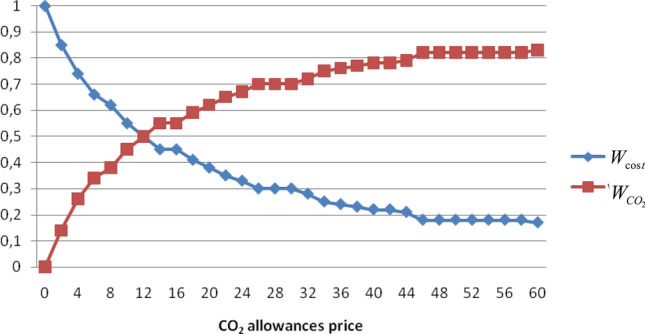
Variation of WFs values in relation to CO_2_ allowances price.

Figure 3 shows that the optimal economic scheduling depends not only on load demand but also it is subject to the greenhouse gases allowances. It can be seen that when the CO_2_ allowances price increases, the CO_2_ weighting factor also increases in order to achieve the optimal economic scheduling. Taking under consideration the impact of CO_2_ allowances price change, the developed software has the ability to automatically read the EU Carbon Permits values from the internet site https://tradingeconomics.com/commodity/carbon and the dispatcher can use this data to rapidly adapt the schedule to the CO_2_ allowances price market changes.

### Case 2. The impact of load variation on the schedule

In this case the importance of total load demand variation on the schedule is examined. The load demand is varied from 1000 to 2000 MW with incremental steps of 50 MW. For all pollutants the allowances cap and the allowances prices remained constant as shown in Table 4.

**Table 4:** Software settings to check the impact of total load variation on the schedule.

P_load_ = 1000–2000 MW	NO_x_	SO_2_	CO_2_
Allowances cap (tn/h)	1	25	55
Allowances price ($/tn)	40	30	20

**Figure 4:**
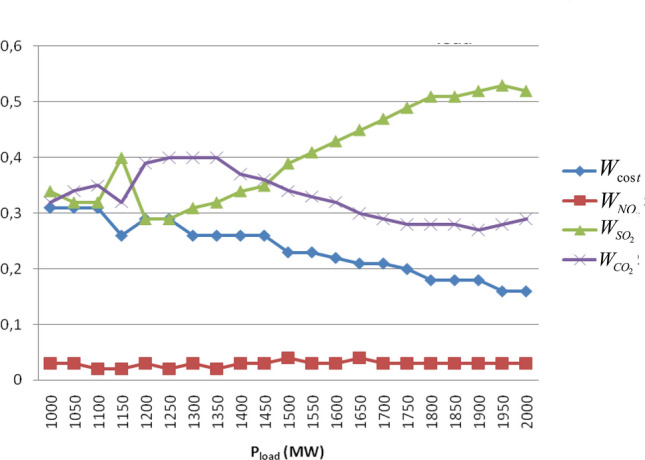
Variation of WFs as a function of Pload when considering allowances for all pollutants.

Figure 4 shows the changes in the weighting factors combinations that have to take place in order the system to function in the most cost-effective manner as the load increases. It can be seen that in different areas of the load range, the dispatcher should focus on lower emissions of different pollutants. This is very crucial for solving cost-effectively the schedule problem including environmental constrains. It is underlined that calculating the values of these weighting factors combinations manually would be almost impossible due to numerous tests for each state that should be carried out. For example, for the resolution of 0.01 which was used in this work, the number of possible tests for each state is 176,000. Executing so many tests manually would be a difficult and time-consuming task. In contrast the developed program calculates the optimum economic schedule both effectively and very fast.

### Case 3. The impact of pollutants allowances on the total cost

In order to assess the impact of pollutants allowances on the total cost and compare it with the conventional scenario we set the pollutants allowances constant (as it can be seen in Table 5), and vary the total load demand in the range of 1000–3000 MW with incremental steps of 100 MW.

**Table 5:** Software settings to check the impact of pollutions allowances on the total cost.

P_load_ = 1000–3000 MW	NO_x_	SO_2_	**CO**_**2**_
Allowances cap (tn/h)	3	25	55
Allowances price ($/tn)	10	10	25

**Figure 5:**
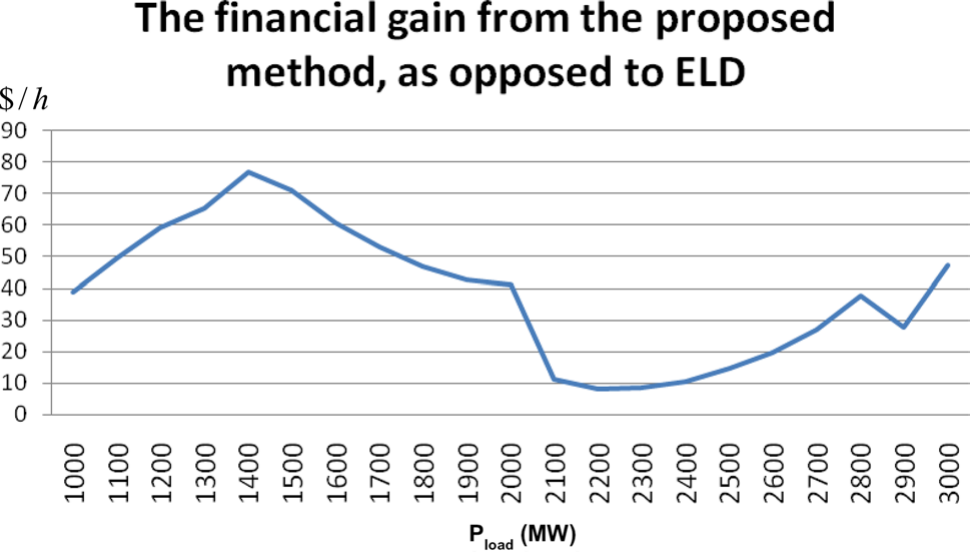
Financial gain variation of proposed method compared to conventional ELD.

Figure 5 indicates that the classic Economic Load Dispatch (ELD), is actually proven to be costlier than the proposed Environmental/Economic Load Dispatch (EELD). Despite the fact that ELD (W_cost_ = 1) schedules the generating units outputs in what is supposed to be the most economic way, it becomes apparent that in power systems where the ETS is implemented, this is not the case. Instead, the more environmentally-friendly schedule turns out to be more profitable through out the entire load range. The financial gain of the proposed EELD in comparison to conventional ELD ranges from 9 to 78$/h depending on the total load demand. This is what makes the proposed method significantly beneficial not only for the environment but also for the power company and the consumers.

## Conclusions

In this paper a new multi-objective optimization approach to EELD problem was presented and for this reason an appropriate Python computer program was developed. This program was applied to a particular thermal power system of six thermal power units considering also greenhouse gases emissions allowances.

It was found that the classic solution of the ELD problem based only on the total cost criterion without consideration of greenhouse gases emissions allowances is obsolete and cost-defective. In the proposed approach the incorporation of emissions allowances in the total cost provides an EELD schedule that is more realistic, cost efficient and environmentally friendly. The problem formulation does not consider the power grid losses and this poses a limitation to its applications in real problems. However, the proposed approach could be the basis for developing a more realistic program incorporating the power system losses for solving efficiently the EELD real problem.

Previous results analysis showed that there is a constant need to calculate the appropriate values of weighting factors. Through the developed software the dispatcher can calculate the schedule of thermal units output either by setting the weighting factors manually or by calculating them automatically according to total load demand and the emissions allowances price. In the manual mode of operation, the dispatcher would have been forced to try many weighting factors combinations (many strategies), in order to find the one that approximately results in minimum cost. Furthermore, for each change in the load or allowances cost, this procedure have to be repeated over and over again. In contrast, the developed software tests all possible weighting factors combinations with resolution 0.01 that would be impossible to be achieved manually and provides the optimum results instantly. The resolution could be increased further up to the limits posed by the calculation capacity of our computer system.

The superiority of the proposed approach compared to established work is its flexibility regarding the pollutants emissions consideration on the solution of the EELD problem. Through the developed program the dispatcher could easily choose the manual mode of operation if a particular environmental issue arise i.e. in case of an urgent need for SO_2_ emissions reduction. In this case the dispatcher will bypass the automatic calculation mode of operation and set the appropriate weighting factors values with more emphasis on SO_2_ emissions. Furthermore, the proposed approach in the automatic mode of operation treats all greenhouse gasses as equally important for the environment without prioritizing CO_2_ emissions and also provides the extra feature for online updating of CO_2_ allowances market prices thus helping the dispatcher to calculate faster the best solution for the EELD problem.

The proposed methodology could potentially be applied to a power plants system where renewable energy sources are considered. In this case, the renewable energy penetration level could be represented by a new weighting factor Wres which can be included in the new problem formulation and the dispatcher will be able to find the optimum solution for the new real EELD problem.

## Data Availability

The corresponding author should be contacted if someone wants to request the data from this study.
